# A Review of the Effects of Maternal and Paternal Obesity on Neurodevelopmental Disorders and Related Neurobiology in Rodent and Human Offspring

**DOI:** 10.1111/obr.70067

**Published:** 2025-12-17

**Authors:** Hannah Chadwick, Victorio Bambini Junior, Neil Dawson, Cheryl A. Hawkes

**Affiliations:** ^1^ Biomedical and Life Sciences Lancaster University Lancaster UK

**Keywords:** ADHD, autism, maternal obesity, neurodevelopmental disorders, paternal obesity, schizophrenia

## Abstract

Obesity is one of the most prevalent health problems worldwide, and global obesity rates continue to rise. Consequently, rates of obesity in expecting mothers and fathers have also increased. The Developmental Origins of Health and Disease (DOHaD) hypothesis postulates that early‐life exposure to adverse environmental conditions contributes to the increased risk of noncommunicable disease later in life. In this context, much work has been done to understand how parental obesity can affect the long‐term health of offspring. In terms of offspring brain health and function, evidence suggests that elevated maternal body mass index (BMI) is associated with an increased risk of autism spectrum disorder (ASD), attention deficit hyperactivity disorder (ADHD), and anxiety disorders, as well as deficits in learning and memory in offspring. Less well characterized is the impact of paternal obesity on the offspring brain. A limited number of studies have reported an association between paternal obesity and altered offspring neurodevelopment, including a higher risk of the offspring having neurodevelopmental disorders such as ASD and ADHD. Mechanisms proposed to underlie these effects include epigenetic modifications, placental changes, and alterations in neuronal protein expression. Here, we review the body of evidence supporting a neurodevelopmental impact of maternal and paternal obesity and associated biological mechanisms. Understanding how parental obesity influences offspring brain function has important implications for the advice given to people trying to get pregnant and expecting mothers, and provides vital insight into the contribution of parental health toward offspring brain health across the life course.

## 1 Introduction

Obesity is one of the most prevalent health problems worldwide, with 14% of adults estimated to be obese globally and another 24% overweight, as measured by body mass index (BMI) [1]. Rates of global obesity are increasing, with prevalence currently highest in industrialized countries such as the USA and UK. For example, in the UK, the 2021 Health Survey for England estimated that 26% of adults are obese, with another 38% being overweight, higher than the global rate of 14% and 24%, respectively [2]. This same survey also found that men are more likely to be obese or overweight than women, with 69% of men being above normal BMI as compared to 59% of women. In the USA, the National Center for Health Statistics reported that from August 2021 to 2023, the prevalence of obesity in adults was 40.3% [3]. Thus, the majority of both UK and USA adults are overweight or obese, and these numbers show an upward trajectory, with recent estimates suggesting that over half of the global adult population will be overweight or obese by 2050 [4].

In line with increasing obesity in the general population, rates of obesity in expecting mothers and fathers have also increased. For example, in 2017, 18% of UK women booking appointments for their first pregnancy were obese, with this rising to 23% in subsequent pregnancies [5]. This has increased since 2007, where first trimester maternal obesity in England was recorded to be 15.6% and has increased markedly since 1989, where first trimester maternal obesity was recorded at 7.6% [6].

Obesity is associated with a long list of well‐established comorbidities, including increased risk of coronary heart disease (CHD), type II diabetes, hypertension, and stroke [7]. Emerging data indicate that being overweight prior to conception also has a negative impact on the long‐term health of children, being associated with an increased risk of asthma, CHD, diabetes, obesity, and premature death in adulthood [8, 9, 10]. The Developmental Origins of Health and Disease (DOHaD) hypothesis suggests that the preconception and perinatal environment impacts the development of the offspring to influence health and disease risk in later life. In particular, fetal adaptations in response to in utero conditions may be maladaptive if there is a mismatch with the postnatal environment [11, 12, 13]. Based on this hypothesis, it is reasonable to suggest that maternal obesity, both through epigenetic changes in the gametes and modification of the in utero and postpartum environment, may have other long‐term health implications for children, and to date, most research has focused on the impact of maternal obesity on offspring outcomes. For example, there is compelling evidence that offspring born to mothers with obesity are more likely to be overweight and to have metabolic disease [14, 15]. Moreover, with respect to the brain, maternal obesity has been linked to altered neurodevelopment in children, leading to a higher risk of developing conditions such as autism spectrum disorder (ASD) and attention deficit hyperactivity disorder (ADHD) [16, 17, 18, 19]. More recently, the Paternal Origins of Health and Disease (POHaD) hypothesis has emerged [20], with data suggesting that the health of fathers can also have long‐lasting effects on their children, caused in part by alterations in the sperm epigenome [21]. While there is less research on this [22], evidence does support an association between paternal obesity and an increased risk of offspring developing ASD and ADHD, alongside impaired cognitive function [23, 24, 25].

This review will outline the current clinical and translational preclinical research characterizing the impact of maternal and paternal obesity on the risk of neurodevelopmental disorders, behavior, and learning and memory in male and female offspring. The suggested mechanisms underlying the observed effects will also be explored, including proposed epigenetic changes that underscore the DOHaD hypothesis.

## 2 The Impact of Maternal Obesity on Offspring Neurodevelopment

Both epidemiological and experimental studies support a link between maternal obesity and impairments in learning, cognition, and behavior in offspring. Alongside this, changes to neuronal and glial cell structure and function have been identified, which may contribute to the observed behavioral changes. Human observational studies examining populations from a range of socioeconomic backgrounds and with a range of obesity‐related exposure measurements, including gestational weight gain (GWG) and prepregnancy BMI, have been conducted. Animal studies have primarily focused on high‐fat diet (HFD) feeding before mating and during gestation and lactation (for maternal studies), and so these will be the primary focus in this review. A summary of these studies is shown in Table 1, with an in‐depth overview of the findings summarized in Tables S1–S3.

**TABLE 1:** A summary of the proposed effects of maternal and paternal obesity on offspring behavior, brain, and epigenetic changes.

	Maternal obesity		Paternal obesity	
	Human	Animal	Human	Animal
Neurodevelopmental disorders (e.g., ASD, ADHD)	↑ Incidence of ASD, ADHD, SCZ, ID	Impaired sociability ↑ hyperactivity	? ‐ potential increase of ASD and ADHD	?
Behavioral and emotional effects	↑ Incidence of anxiety, depression, behavioral problems	↑ Anxiety‐like behavior in OFT and EPM ↑ Depressive behavior in FST	? ‐ potential link to increased anxiety and depression	↑ Anxiety‐like behavior in OFT and EPM
Learning and memory	↓ IQ ↓ Cognitive performance ↓ Memory	↓ Memory in OA task and NORT ↓ Reversal learning	? ↓ IQ ? ↓ Cognitive performance ? ↓ Memory	↓ Spatial memory ↓ Short‐term memory in the NORT
Epigenetics	↑ Methylation patterns changes	↑ Methylation patterns changes ↑ ncRNA changes ‐miRNA ↑ Chromatin remodeling via histone modification	↑ Methylation patterns changes ↑ ncRNA sperm content changes—tsRNA, miRNA ↑ Sperm histone modifications	↑ ncRNA sperm content changes—tsRNA, miRNA
Brain changes	↓ BDNF ↑ Apoptosis and neurogenesis dysregulation ↑ Inflammation	↓ BDNF ↑ Pro‐inflammatory cytokines ↑ Microglial activation ↓ apoptosis ↓ neuronal differentiation	—	↓ BDNF ↑ Neurogenesis dysregulation
Placenta	↑ Macrophage activation ↑ Cytokine activation ↓ BDNF	↓ Proliferation and labyrinth thickness ↑ Macrophage activation ↑ Cytokine activation	—	↑ Vascularization changes ↑ Placental methylation and gene expression changes
Offspring sex differences	Males more strongly affected	Males more strongly affected	? ‐ Females more strongly affected	? ‐ Females more strongly affected

**↑,** denotes increase; ↓, denotes decrease; ?, denotes a lack of clear evidence or no reported evidence; ADHD, attention deficit hyperactivity disorder; ASD, autism spectrum disorder; BDNF, brain‐derived neurotrophic factor; ID, intellectual disability; EPM, elevated plus maze; FST, forced swimming test; OA, odor aversion; OFT, open field test; NORT, novel object recognition test; SCZ, schizophrenia.

### 2.1 Neurodevelopmental Disorders

#### 2.1.1 Autism Spectrum Disorder

Population‐based studies have reported positive associations between higher GWG and an increased risk of ASD in children, independent of prepregnancy BMI [26, 27, 28]. Multiple studies have also reported a link between high maternal BMI (≥30 kg/m^2^) during pregnancy and an increased risk of ASD in their children [29, 30, 31, 32, 33, 34, 35], including in a recent meta‐analysis (OR = 1.41) [16]. Interestingly, maternal underweight has also been proposed as a risk factor for ASD [36, 37, 38] while other studies have reported no association [26, 39] between maternal weight during pregnancy and offspring ASD risk. This relationship may also be mediated by other maternal factors, as the study by Getz et al. [37] found an increased risk of ASD only in children of underweight mothers who were also over 30 years old. These studies suggest that a U‐shaped relationship between maternal weight and offspring ASD risk may exist, with both maternal overweight and underweight increasing risk.

Animal studies have primarily focused on characterizing translational behaviors relevant to ASD, including impaired sociability. In mice, offspring of mothers fed a HFD before mating, and during gestation and lactation, demonstrate impaired social behavior as compared to offspring born to mothers fed a standard control diet [40]. A recent study by Zilkha et al. [41] found reduced sociality, increased aggression, and cognitive rigidity in male mice born to HFD‐fed dams, compared to those born to mothers fed a low‐fat diet. Similar findings have also been reported in male rats exposed to a HFD during pregnancy and lactation, in association with altered expression of ASD risk genes such as *Nlgn3* and *Shank1* in the prefrontal cortex of offspring [42]. Overall, these studies support autism‐like social behavioral deficits in rodent offspring exposed to a HFD during development and lactation, with molecular changes in pathways well‐established in ASD risk. However, further work is needed to elucidate the specific behavioral domains altered in mice, the mechanisms involved, and how these map onto alterations seen in children with a diagnosis of ASD.

#### 2.1.2 Attention Deficit Hyperactivity Disorder

Human observation studies have also linked maternal prepregnancy BMI with an increased risk of ADHD, characterized in children aged 5–8 years [43, 44, 45]. It has been suggested that this effect may be mediated by impaired executive function [46]. However, a sibling comparison study conducted by Chen et al. [47] found that the association between maternal BMI during pregnancy and offspring ADHD was lost when adjusting for sibling relationships, suggesting that positive associations may be confounded by genetics or other shared environmental factors.

Although few rodent studies have been conducted in the context of ADHD, Fernandes et al. [48] found that male offspring of obese dams were more active in behavioral tasks than control mice and showed signs of hyperactivity. Similar results were also reported by Kang et al. [49], who observed hyperactive behavior in male offspring of HFD‐fed dams. While these studies suggest an increase in behaviors potentially relevant to ADHD in the offspring of obese dams, more work is needed to more firmly support the association of maternal obesity and increased risk of ADHD in their offspring. In addition, a broader scope of ADHD‐relevant phenotypes in rodents, such as attentional abilities and impulsivity [50], should be assessed to further establish the alignment of the rodent models with the human condition. Moreover, given the reported mediating effect of executive function deficits on ADHD risk [46], characterizing these cognitive domains would be of particular interest.

#### 2.1.3 Schizophrenia

Schizophrenia (SCZ) is a severe psychiatric disorder that is neurodevelopmental in origin [51]. An increased risk of SCZ has been reported in the offspring of mothers living with obesity in a limited number of human studies [52]. An early study found that the risk of SCZ was three times higher in 30‐ to 38‐year‐old offspring of mothers with a preconception BMI >30 kg/m^2^ [53]. However, some overweight mothers included in the study were given amphetamines prior to or during pregnancy, which may have skewed the outcomes due to neuroanatomical and cognitive changes observed in children that have experienced prenatal exposure to amphetamines [54, 55]. Nevertheless, a more recent longitudinal study of 68,571 mother–child dyads also found that prepregnancy maternal BMI >35 kg/m^2^ increased the likelihood of offspring developing SCZ spectrum disorders in adulthood by 2.8‐fold [56]. High maternal BMI in early and late pregnancy was also associated with an increase in SCZ risk, with an increased risk of 24% in early pregnancy and 19% in late pregnancy per maternal BMI point [57]. Thus, current evidence supports a positive association between high maternal BMI and increased risk of SCZ in adult offspring. However, as in other severe neuropsychiatric disorders, caution must be taken in relation to the direction of causation between maternal obesity and SCZ risk, particularly given the increased BMI reported in individuals with a SCZ diagnosis [58], potentially resulting from the obesogenic and metabolic side effects of antipsychotic medications [59]. Thus, controlling for maternal psychiatric diagnosis, medication status, and underlying genetic contributions, through Mendelian randomization or sibling‐matched designs is important, particularly given the significant genetic contribution to neuropsychiatric and neurodevelopmental disorder risk [59]. These potential confounds have not always been controlled for [52], although studies controlling for maternal psychiatric diagnosis [53] and genetic contributions [56], do support an association between high prepregnancy maternal BMI and offspring SCZ risk. These limitations also apply to studies on the association of paternal obesity with SCZ and with other neuropsychiatric disorders [60]. Despite these epidemiological associations, there has been very little preclinical rodent research into mechanisms by which maternal obesity leads to an increased risk of SCZ. However, the previously reported effects on hyperactivity [48, 49] may be relevant, as this is often considered a measure of dopaminergic dysfunction that is associated with the positive symptoms of the disorder [61]. Clearly, further research is required in rodent models to elucidate the mechanisms by which maternal obesity increases SCZ risk, including elucidating the SCZ‐relevant behavioral domains that are most susceptible.

### 2.2 Affective Disorders

#### 2.2.1 Anxiety and Depression

Maternal obesity has also been linked to increased incidence of anxiety and depression in offspring. One longitudinal study found that children of mothers who were obese during pregnancy had significantly more clinician visits for mental health reasons, including mood and anxiety disorders, when monitored from birth to 18 years old [62]. A similar 2012 Australian cohort study looked at the incidence of affective disorders, including major depressive disorder (MDD) and dysthymic disorder, in children from birth to age 17. They found that the children of mothers who were overweight or living with obesity before pregnancy had a higher risk of being diagnosed with an affective disorder such as depression between ages 5 and 17 [63].

Studies in mice also support the regulation of affect by maternal obesity, with one study reporting increased anxiety‐like behavior in 3‐month‐old offspring born to HFD‐fed mothers [64]. Another mouse study also found increased anxiety‐like behaviors in 12‐month‐old offspring of HFD dams, although this was not evident when the offspring were 3 months old, supporting a potential neurodevelopmental role in the emergence of this phenotype [65]. Adolescent and young adult offspring of HFD‐fed rats also showed an increased anxiety‐like response compared to control animals across multiple behavioral tasks, including in the elevated plus maze (EPM) and open field test (OFT) [66, 67]. This was associated with increased neuroinflammation, including altered expression of anti‐ and pro‐inflammatory cytokines, in the hippocampus of adult HFD offspring [66]. Similar results were also seen in another mouse study, which found increased anxiety‐like behavior in adult offspring of HFD‐fed mothers, alongside microglial activation in the hippocampus [68]. Abnormal glutamate homeostasis in the amygdala has also been reported in mouse offspring of HFD mothers, again associated with increased anxiety‐like behavior in 4‐month‐old animals [69]. In addition to these rodent experimental studies, work has been undertaken in primates to ascertain the conservation of the affective effects of maternal obesity in offspring in higher vertebrate species. Interestingly, increased microglia numbers in the amygdala have been observed in juvenile nonhuman primate offspring as a function of maternal diet and adiposity [70], supporting a conserved role for neuroinflammation and elevated anxiety‐like behavior in primates. Other nonhuman primate models of maternal HFD have also reported increased anxiety‐related behaviors in the offspring, alongside disturbances in the serotonergic system in offspring at 4 and 11 months of age [71, 72].

Mice and rats born to mothers fed a HFD also exhibit higher levels of depressive‐like behavior, as measured by the forced swim test at postnatal days 34, 69, and 90 [42, 73]. This phenotype was associated with an imbalance in the excitatory–inhibitory (E–I) neurotransmitter systems, indicated by an upregulation of excitatory glutamatergic neuronal markers and a downregulation of inhibitory GABAergic neuron markers in the frontal cortex of young adult offspring [42]. Therefore, the available preclinical data not only support an increased risk of anxiety and depressive‐like behavior in the offspring of mothers who were overweight or living with obesity but also identify changes in cellular brain structure and neurotransmitter system function that may contribute to these outcomes.

### 2.3 Behavioral and Cognitive Impacts, Including Intellectual Disability

In addition to depression and anxiety, an increased prevalence of general behavioral problems has also been reported in children of mothers who were obese before pregnancy, including internalizing and externalizing problems [25, 74, 75, 76]. Menting and colleagues [77] reported a 30%–70% increase in behavioral problems in 5‐year‐old children with maternal prepregnancy overweight/obesity. By contrast, Brion et al. [78] found no association of maternal prepregnancy weight with children's behavioral problems, nonverbal skills, or attention issues across two cohorts. However, some data in the study depended solely on maternal reporting, which may have led to bias as compared to other studies that used teacher reporting or more objective assessment methods.

Studies have also reported an association between maternal weight and cognitive deficits in offspring, including a delay in childhood mental development [79]. Excess GWG has been associated with intellectual development disorder (IDD), with a GWG of greater than 25 kg associated with increased risk of offspring IDD. However, this effect was only seen in mothers that had a BMI of 25 or more in early pregnancy [80]. With respect to prepregnancy maternal weight, a 2017 systematic review and meta‐analysis of 15 human studies concluded that prepregnancy obesity negatively affected offspring cognition, and also had a small negative effect on general intelligence [81]. Using a range of cognitive assessments, other studies have found that children of mothers with a higher prepregnancy BMI achieved lower test scores [81, 82, 83, 84], and had a lower IQ than children of normal weight mothers [85, 86, 87, 88, 89]. The strength of this interaction may also depend on offspring age. Basatemur et al. [83] reported a small negative correlation between maternal BMI during pregnancy and child cognitive performance at ages 5 and 7, which increased in magnitude as the children grew older. However, as noted by the authors, this may have been influenced by using different cognitive tests at the different ages. A population study by Mann and colleagues [91] of 78,675 mother–child pairs found an association between prepregnancy maternal obesity and ID in children. This risk was even larger for children of mothers living with class III obesity (BMI >40).

In animal studies, White and colleagues [92] found no differences between male mouse offspring born to mothers fed a HFD or control diet before mating and during gestation and lactation in the acquisition or retention of spatial memory in the Morris water maze (MWM). However, offspring of mothers fed a HFD that were themselves exposed to a HFD after weaning showed deficits in memory retention as well as evidence of brain inflammation and oxidative stress, suggesting that the maternal HFD may have “sensitized” the offspring to the detrimental effects of a HFD. By contrast, other rodent studies have reported a direct effect of maternal HFD on cognitive deficits in the offspring [93]. One study testing memory in adult rats, using an odor‐based aversion task, found impairments in memory and corresponding morphological changes in hippocampal and amygdala neurons, including dendritic shrinkage in hippocampal cornu ammonis 1 (CA1) and basolateral amygdala pyramidal neurons [94]. Memory deficits have also been reported across adolescent, young adult, and adult rats born to HFD‐fed mothers, as assessed in the novel object recognition test (NORT), in multiple studies [94, 95, 96]. This was accompanied by alterations in glutamatergic signaling, including downregulated NMDA receptor (NMDA‐R) subunit levels in the prefrontal cortex and hippocampus [94, 96, 97]. Another study focused specifically on cognitive flexibility, as indicated by reversal learning in an operant conditioning task, found impaired reversal learning in rats born to obese dams. This was associated with impaired dopamine homeostasis and leptin signaling in the striatum, supporting a role for dysfunction in these signaling systems induced by maternal HFD exposure [99].

Interestingly, the impact of maternal obesity on offspring cognition in rodents also seems to be influenced by offspring age. Tozuka et al. [100] found that spatial learning in the Barnes maze was impaired in 4‐week‐old animals born to dams fed a HFD, while performance in 11‐week‐old animals was not. This was associated with decreased levels of brain‐derived neurotrophic factor (BDNF) in the hippocampus of young mice, which has an established role in promoting neuronal plasticity and learning [101]. However, the opposite was found in another study, where performance in cognitive tests declined to a greater extent with age in offspring from HFD dams than in controls [102]. While this second study aligns with the observations made by Basatemur et al. in humans, it is clear that more research is needed to clarify the modulatory influence of offspring age in the relationship between maternal obesity and offspring cognition.

### 2.4 Sex Differences in the Behavioral and Cognitive Impact of Maternal Obesity

Interestingly, the impact of maternal obesity on offspring behavior and cognition appears to be sexually dimorphic. In a human cohort study, an association between maternal prepregnancy overweight and obesity and lower IQ was observed in 7‐year‐old male, but not female, children [87]. Male children of mothers with prepregnancy obesity were also reported to have a significantly lower Psychomotor Development Index score at 3 years old compared to mothers with a normal prepregnancy BMI, but this association was not apparent in female children [85]. Similarly, in rodent studies, MWM performance was reported to be impaired in male, but not in female, offspring born to mothers fed a HFD [103]. This has also been seen in the NORT, with male offspring of HFD‐fed mothers showing greater impairment than females [103, 104], again supporting the increased sensitivity of males to maternal obesity in relation to cognitive impacts.

By contrast, females may be more sensitive to the anxiogenic impact of maternal obesity. In rodent studies of anxiety‐like behavior, Sullivan et al. [71] found that female offspring of HFD‐fed mothers showed increased latency to touch unfamiliar objects, while this was not observed in male littermates. Instead, males from HFD‐fed mothers showed increased aggression compared to male control offspring. This study suggests that the impact of maternal obesity on anxiety‐related behaviors may be sexually dimorphic. Alternatively, it may be that the impact of maternal obesity on offspring anxiety is displayed differentially between male and female rodents, with males becoming more aggressive and females more withdrawn. Indeed, a 2021 systematic review of rodent anxiety studies concluded that the impact of maternal HFD on male offspring is stronger and more consistently reported than in female offspring. However, as twice as many male mice were included across the studies reviewed than females, mirroring the well‐recognized sex bias in biological literature [106], the reported sexual dimorphism of maternal obesity may also be due to the discrepancy between sex sample sizes [107].

A diverse range of biological measures also supports a potential sexual dimorphism in the impact of maternal obesity on the offspring brain. A study of 88 children aged 7–11 years old found that boys of mothers with a high prepregnancy BMI had significantly lower hippocampal volume than boys born to lean mothers, but this relationship was not observed in daughters [108]. Interestingly, in rodents, sex‐based differences have also been reported in terms of the impact of maternal HFD on hippocampal gene expression, with genes for epigenetic regulators and oxytocin expression more impacted in males than in females [69, 108]. Microglial alterations have also been observed to be sexually dimorphic, with adolescent male mouse offspring showing more changes in gene expression, reduced microglial receptor expression, and increased microglial interaction with astrocytes when compared to female offspring of HFD‐fed mothers and control offspring [110]. While emerging clinical and preclinical data support a sexually dimorphic response to maternal obesity in the offspring, further research is clearly needed to clarify the potential sexual dimorphisms that may exist in relation to the cognitive, affective, and behavioral impacts of maternal obesity on the offspring and the mechanisms involved.

## 3 The Impact of Paternal Obesity on Offspring Behavior, Cognitive Function, and Neuronal and Glial Expression

Comparatively, the effects of paternal obesity on the neurodevelopment of offspring have been far less researched and so the evidence base is much more limited. There is strong emerging evidence that paternal obesity can influence offspring neurodevelopment, cognition, and mental health, independently of maternal health [17]. However, there is a need for much broader study in this area, including the need to study effects in ethnic and socioeconomic under‐represented populations.

### 3.1 Neurodevelopmental Disorders

#### 3.1.1 Autism Spectrum Disorder

A 2014 study investigating 92,909 children ranging from 4 to 13.1 years old reported an increased risk of ASD (OR = 1.73 for autistic disorder, OR = 2.01 for Asperger disorder) with elevated paternal BMI. Interestingly, this study also found the paternal association to be stronger than the maternal influence [23]. Deficits in sociability in the offspring of fathers with obesity, similar to those seen in individuals with ASD, have also been reported [111]. Preconceptual paternal obesity has also been associated with the increased risk of children failing the personal–social domain section of the Ages and Stages Questionnaire (ASQ) between ages 0 and 36 months, supporting an impact on social–emotional development [111]. However, a more recent meta‐analysis comprising three studies reported no association between paternal weight and ASD based on the pooled OR (OR = 1.28, 95% CI: 0.94–1.74), with the authors recognizing the need to increase the evidence base in this area of research [16, 23, 28, 39]. Also, research using paternal obesity as a confounder found no independent association between paternal BMI at the time of pregnancy and autistic traits in their offspring at 19–20 years old [112]. Future studies into the topic are needed to address the disparity between these studies, with characterizing at a range of offspring ages required to clarify any developmental effects.

#### 3.1.2 Attention Deficit Hyperactivity Disorder

ADHD risk has also been linked to paternal BMI. A recent study found that preconceptual paternal obesity increased the risk of ADHD twofold compared to children born to normal weight fathers [24]. An earlier study also reported that 7‐year‐old children had a 37% higher risk of an abnormal hyperactivity score if their father had preconceptual obesity, compared with children of fathers with a BMI <25 kg/m^2^ [25]. However, studies in which paternal BMI was included as a confounder for maternal prepregnancy BMI have reported no relationship between paternal weight and offspring ADHD risk [45]. Therefore, more research is essential to allow more concrete conclusions to be drawn.

#### 3.1.3 Schizophrenia

To the best of our knowledge, there are currently no published data on the potential association of SCZ risk with paternal obesity. This is particularly surprising given the association of maternal obesity with SCZ and the observation that paternal factors such as increased age are associated with an increased SCZ risk [113]. The influence of paternal age is thought to be mediated by de novo mutations and epigenetic alterations in the sperm [114], two mechanisms that are also influenced by paternal obesity, as discussed later in this review. This includes at least some mechanistic overlap with the epigenetic changes present in the sperm of HFD animals, which include altered gene methylation in growth factor and immune regulatory pathways [115].

Unfortunately, little preclinical work has also been conducted into the potential impact of paternal obesity on ASD, ADHD, and SCZ‐relevant phenotypes in rodents, and the disease‐relevant biological mechanisms involved, although there are reported changes in cognitive and affective processing that may be relevant to these disorders (discussed below).

### 3.2 Behavioral and Emotional Disorders

To date, the impact of paternal HFD on offspring anxiety has been understudied. In a human study, paternal obesity was found to increase the risk, by 84%, of children showing behavioral difficulties that impacted their daily life at 7 years old [25]. The potential relationship between paternal obesity and the likelihood of the offspring developing affective disorders, such as MDD and anxiety disorders, has also not been reported. Given the observations and relatively strong effects reported in the maternal literature, this certainly warrants further investigation. Indeed, although also limited, the available rodent data support the potential affective impact of paternal obesity. Korgan et al. [116] found that 35‐ to 42‐days‐old offspring born to HFD‐fed fathers spent less time in the center zone of the OFT and spent less time in open arms of the EPM, supporting an increased anxiety‐like phenotype in these animals.

### 3.3 General Intelligence and Learning and Memory

A few studies have also reported an association between paternal prepregnancy BMI and offspring cognition. One such study found that paternal BMI was associated with a 0.26‐point reduction in IQ per unit increase in paternal BMI, in 5‐year‐old children [117]. Coo et al. [118] also observed a negative relationship between paternal obesity and child IQ, although the association with maternal obesity was stronger.

Mouse models have also explored the association between paternal obesity and learning and memory. One study found that first‐generation offspring born to fathers fed a HFD performed worse than control offspring in the MWM task at 8 weeks old and showed a reduction in BDNF levels in the hippocampus, suggesting impaired hippocampal‐dependent learning and memory, associated with reduced neurotrophic signaling [119]. Another study in mice using the NORT found that short‐term (2 h), but not long‐term (24 h), memory was impaired in offspring of HFD fathers at 4 months old [95]. This was associated with the downregulation of glutamatergic NMDA‐R in the hippocampus and frontal cortex, as well as reduced expression of genes involved in synaptic plasticity and learning and memory [95]. Thus, the availability of preclinical data supports an impact of paternal obesity on learning and memory through the perturbation of well‐established mechanisms underlying these processes.

### 3.4 Sex Differences in Behavioral and Cognitive Impact of Paternal Obesity

It has not yet been elucidated whether the impact of paternal obesity on offspring brain development and function is also subject to sexual dimorphism, as supported by observations in maternal obesity. The general lack of paternal obesity research, as well as the fact that studies frequently test only one sex, with a strong male bias in the literature, have led to a lack of evidence in this research space that prevents comparison between the sexes to characterize any dimorphic responses. Future studies should include both biological sexes so that any sexually dimorphic impact of paternal obesity on offspring health can be elucidated.

## 4 Proposed Mechanisms of Effect

**FIGURE 1:**
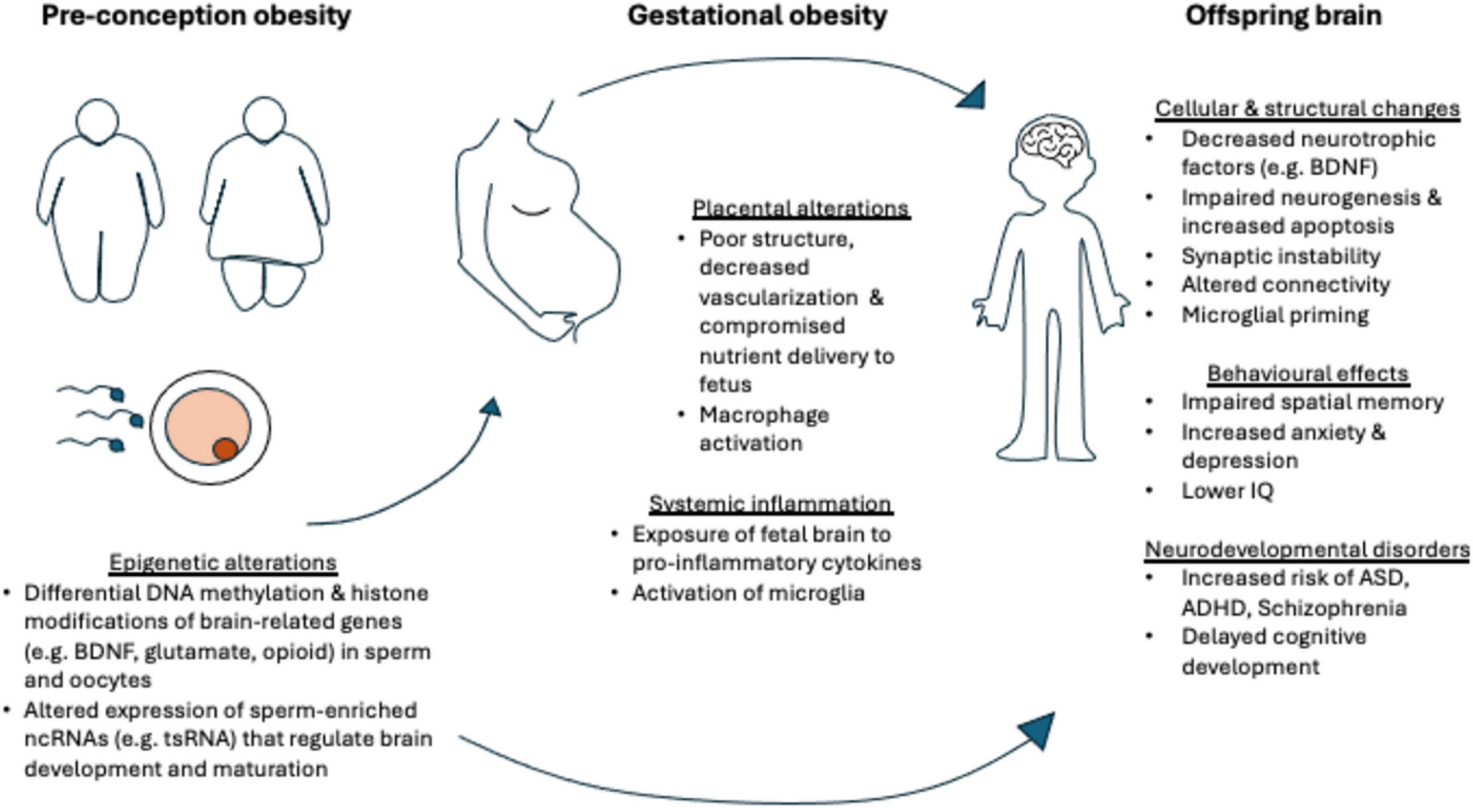
Putative mechanisms by which parental obesity affects the offspring brain. Preconception obesity in mothers and fathers is associated with epigenetic alterations of brain‐related genes in germ cells. In addition, during pregnancy, placental abnormalities in mothers with obesity, in conjunction with fetal exposure to obesity‐associated inflammation, can predispose the offspring brain to cellular and structural changes. These changes may underlie neurodevelopmental, cognitive, and behavioral deficits seen in the offspring. Note that evidence from paternal studies is currently limited and offspring outcomes are still debated.

Ongoing research is characterizing the molecular and cellular mechanisms underlying the intergenerational effect of parental health on the offspring. Most research has focused on obesity‐induced epigenetic alterations that are passed onto the offspring, resulting in changes to the expression of genes involved in metabolism and neurodevelopment. There is also increasing evidence for changes in placenta structure and function and neuronal function, which may underlie the observed behavioral changes. An overview of the proposed mechanisms by which maternal and paternal obesity influence offspring brain structure and function is shown in Figure 1.

### 4.1 Maternal Mechanisms of Impact

#### 4.1.1 Epigenetic Mechanisms

Epigenetics describes heritable changes to DNA that do not involve changes to the genetic code. These include DNA methylation, histone modification (through chemical alterations or different histone variants), and noncoding RNAs (ncRNAs). DNA methylation describes the transfer of methyl groups via DNA methyltransferases onto (primarily) cytosine residues that precede guanine residues, known as CpG sites, in DNA [120]. When these CpG sites are located within gene promoter regions, this methylation typically silences gene transcription. Changes to histones, either through histone variants or chemical changes including acetylation, phosphorylation, methylation, or ubiquitination, lead to alterations in the strength of DNA–histone interactions, and thus the packaging of DNA [121, 122]. This can cause DNA sections to be packaged more tightly, as heterochromatin, placing genes into a less actively transcribed state, or to be more loosely packaged, as euchromatin, with genes more actively transcribed [123]. Finally, ncRNAs produced from noncoding sections of DNA are involved in gene regulation and silencing [124]. The main types of ncRNAs include micro (miRNA), small interfering (siRNA), small nuclear (snRNA), piwi‐interacting (piRNA), transfer RNA‐derived small RNAs (tsRNA), and long (lncRNA) [124]. All have varied lengths, structures, and functions, but overall they work to modulate gene expression through transcriptional or posttranscriptional modifications and by influencing chromatin remodeling [125].

There is current debate around the heritability of epigenetic changes and whether they persist in subsequent generations. To be inherited, they must be both maintained in germline cells and persist after the global methylation remodeling that occurs after fertilization [126]. There are currently two main hypotheses of epigenetic inheritance. The first suggests that some DNA regions escape reprogramming and maintain methylation states in the zygote [127, 128]. The second proposes that DNA methylation and histone modifications are erased in fertilized cells, but that prefertilization patterns are reproduced in offspring DNA in response to secondary signals, including ncRNAs and transcription factors [129]. In human studies, the collection of cord blood from newborns, as a proxy for the somatic tissues, found that the methylation status of 481 CpG sites was different between children born to normal weight versus mothers who were overweight or living with obesity, with several of these methylated genes known to be involved in energy balance and metabolism [130]. In addition, a study involving siblings born before and after maternal bariatric gastrointestinal bypass surgery found 5698 genes that were differentially methylated, mainly involved in inflammatory, immune, and metabolism pathways [131]. These studies support the potential impact of maternal BMI on offspring health via modification in DNA methylation, perhaps contributing to the higher risk of obesity seen in children born to mothers with obesity before pregnancy [130, 132].

Alterations in the methylation status of genes involved in the serotonin pathway, measured in newborn cord blood, are also associated with high maternal prepregnancy BMI and GWG [133], suggesting an additional epigenetic mechanism by which maternal obesity may impact the offspring brain. This is also supported by observations from animal studies, with maternal HFD shown to alter the expression of genes implicated in ASD in the frontal cortex of male rat offspring such as *SHANK1*, *SETD1B*, and *ANKRD11* [134], which have roles in neuron differentiation [135], synaptic scaffolding [136], and control of gene expression [137]. The same study also found concomitant differences in DNA methylation in the frontal cortex and hippocampus that also support perturbed brain function [134]. Differences in DNA methylation patterns in the brains of offspring were also seen between mice born to mothers fed a HFD versus a control diet, with hypomethylation in the promotors of genes involved in dopamine and opioid signaling detected [138]. In addition, macaque infants from obese mothers also showed differentially methylated DNA patterns in the brain [139]. This included differential methylation of genes involved in glutamatergic synapses, angiogenesis, cellular adhesion, and cellular signaling. Overall, emerging evidence suggests that increased maternal BMI alters the methylation status of genes that play key roles in regulating brain and neurotransmitter system function, which may contribute to the underlying behavioral changes.

Changes to ncRNA expression have also been detected in the offspring of mothers with obesity. However, this has been researched in the context of the inheritance of obesity risk rather than in terms of the neurodevelopmental and cognitive impact. Nevertheless, the available data support altered ncRNA expression in the offspring brain. A recent mouse study found the microRNA miR‐505‐5p was overexpressed in the hypothalamus of offspring born to obese mothers, and this effect persisted through to adulthood [140]. Target genes of this miRNA are involved in neuronal fatty acid uptake and metabolism, and thus overexpression led to altered neuronal fatty acid sensing and an increase in feeding behavior [140]. ncRNA expression has also been linked to dysregulated lipid metabolism in the liver of HFD offspring, potentially leading to increased obesity risk. This has been seen in mice through dysregulation of lncRNAs [141] as well as miRNAs [142, 143]. miRNAs have also been identified as potential therapeutic targets for obesity through exploiting their roles in metabolism. An experiment using a mimic of miRNA‐21, an miRNA that is highly expressed in individuals with obesity [144], found that therapeutic application delayed the onset of obesity in mice fed a HFD [145].

Chromatin remodeling via histone modification has also been seen in maternal HFD offspring, with male HFD offspring showing increased histone acetylation in the transcriptional start site of the oxytocin receptor gene in the hippocampus [109]. This is interesting, given the role of oxytocin as a neurotransmitter and its involvement in social behavior, with aberrant oxytocin signaling implicated in the etiology of various neurodevelopmental disorders including ASD, ADHD, and SCZ [146, 147]. Thus, histone modifications may contribute to the social deficits reported in maternal HFD offspring [40]. Another study in mice also found an impact of maternal HFD on histone acetylation in the embryo, implicating the abnormal regulation of genes involved in embryonic development and an associated increased incidence of neural tube defects [148].

#### 4.1.2 Inflammation

Fetal development is a critical period that is sensitive to adverse environmental conditions, such as those induced by an obesogenic diet. Obesity is associated with chronic low‐grade inflammation [148], and during pregnancy, this can result in a pro‐inflammatory in utero environment. Correlations have been shown between prepregnancy obesity and increased plasma pro‐inflammatory cytokines, including TNF‐α and interferon‐gamma (IFN) [150], and increased monocyte activation [151] during gestation. Interestingly, epidemiological studies investigating cytokine levels during pregnancy with the development of SCZ found an association with pro‐inflammatory cytokines TNF‐α [152] and IL‐8 [153], with others postulating ASD may also share similar etiological roots regarding exposure to inflammation during development [154].

Fetal brain microglia have been reported to be “primed” by a maternal HFD, making the offspring more sensitive to subsequent immune challenge [110, 155]. In addition, pro‐inflammatory cytokine [66] and microglial [68] activation have been reported in the hippocampus [66, 68] and amygdala [70] of HFD offspring mice displaying an anxiety‐like phenotype. Interestingly, microglial activation was found to be sexually dimorphic in one study, with male offspring showing altered expression of inflammatory and microglial regulatory genes [110], which were not seen in female offspring.

The interplay between perinatal inflammation, offspring neuroinflammation, and behavioral effects is interesting and may represent a strong mechanistic hypothesis for the neurodevelopmental effects of maternal obesity. However, the exact mechanisms remain unknown and require more research.

#### 4.1.3 Neuronal and Brain Changes

As discussed previously, maternal obesity alters brain structure, neuronal function, and the function of microglia. However, the exact mechanisms involved are still unknown.

One key mechanism may be the epigenetic modification of the BDNF gene, resulting in dysfunctional BDNF signaling. BDNF is involved in the growth and survival of neurons, neuronal plasticity, and learning and memory [101]. In rat models of maternal high fructose feeding, increased methylation of the BDNF promoter and reduced levels of hippocampal BDNF were observed, along with impaired spatial memory [156, 157]. The prolonged impact of this mechanism is supported, as this hypermethylation was still present when rats were 60 days old. Similar effects have also been reported in mice, with BDNF mRNA and protein levels reduced in the hippocampus of 21‐day‐old offspring of obese dams [100]. Interestingly, in a preclinical intervention study, folate administration attenuated this maternal HFD‐induced BDNF hypermethylation, with a parallel recovery in cognitive performance and anxiety‐like behavior compared to nonsupplemented HFD offspring, further supporting the importance of this mechanism [158]. Importantly, decreased BDNF levels have been linked in meta‐analyses to SCZ [159] and depression [160]. In addition, higher levels of peripheral BDNF have been reported in children diagnosed with ASD [161, 162]. Interestingly, BDNF has also been identified as a potential candidate for the sex biases seen in many neurodevelopmental disorders such as ASD due to the regulation of BDNF by estrogen [163]. Therefore, perturbations in BDNF signaling, mediated by epigenetic changes, may be a key mechanism through which maternal obesity impacts neurocognitive outcomes for the offspring and may contribute to some of the observed sex differences.

BDNF signaling influences neuronal apoptosis and proliferation [164], so epigenetic regulation of BDNF expression may also have a role in promoting the dysregulation of these mechanisms in response to maternal obesity. Thus, the dysregulation of apoptosis and neurogenesis has been suggested as another mechanism by which maternal diet alters the offspring brain. In humans, a preliminary study using amniotic fluid samples from women with obesity in the second trimester of pregnancy found altered expression of 205 genes compared to samples from normal weight women, including the downregulation of genes involved in apoptosis pathways in the cerebral cortex [165]. A mouse model of maternal obesity also found changes in the proliferation of neural progenitors, reduced apoptosis, and decreased neuronal differentiation in the dentate gyrus of the hippocampus at gestation day 17 [166]. Synaptic instability and increased elimination of dendritic spines have been linked to HFD consumption prior to mating in a mouse model [167]. Synaptic pruning, the targeted removal of synapses throughout development, is dysfunctional in neurodevelopmental disorders, including ASD and SCZ [168], with a failure of pruning and increased synaptic density seen in ASD and loss of synaptic density supported in SCZ. In addition, impaired neurogenesis is also supported in these disorders [169, 170]. Thus, alterations in neurogenesis and synaptic pruning may also be key mechanisms through which maternal diet impacts offspring behavior and neurodevelopmental disorder risk. Importantly, both neurogenesis and synaptic pruning are regulated by cells of the immune system (microglia), with these cells proposed to have a key role in neurodevelopmental disorders [171, 172], providing a potential additional mechanistic link to the pro‐inflammatory environment seen in maternal obesity.

Morphological changes to the offspring brain have been associated with maternal obesity. In a recent human study, a positive association was discovered between prepregnancy maternal BMI and mean diffusivity, a measure of water diffusion through the tissue, in the hippocampus, potentially indicating microstructural changes in the offspring hippocampus [173]. Sex differences in hippocampal volume have also been reported, as mentioned previously, with male children of mothers with a high prepregnancy BMI showing a reduced hippocampal volume compared to controls, an association not seen in female children [108]. Reduced cortical thickness and hippocampal volume were also found in mouse offspring of HFD mothers [174], supporting the associative observations in human studies. A 2024 review of 15 human studies studying the impact of maternal prepregnancy obesity on offspring brain structure found that significant differences were reported in 12 of these studies [175]. Results were most consistently found in the prefrontal cortex and limbic system, with changes in volume, diffusion rate, and connectivity reported [175]. These morphological changes to offspring brain structure and function, as a consequence of maternal obesity, may underlie changes in behavior, cognition, and memory seen in the offspring due to the well‐established roles of these neural systems in executive function and learning and memory [176].

#### 4.1.4 Placental Alterations

Obesity‐induced placental alterations have also been explored as an influential mechanism on offspring health, considering the key role the placenta has in supporting fetal development and as the point of exchange between mother and child. A mouse model of maternal obesity observed decreased proliferation and labyrinth thickness, with increased inflammation, indicated by increased macrophage activation and cytokine gene expression, in the placentas of HFD‐fed mothers [177]. Other studies have reported similar findings [155, 178]. Interestingly, ASD has been associated with increased placental inflammation, which may contribute to the increased incidence associated with maternal obesity [16, 179]. Within the placenta of mothers with obesity, there are also reduced BDNF levels, which may also influence fetal programming and offspring outcomes [180].

#### 4.1.5 Sex Differences

The mechanisms above may have a role in mediating some of the sex‐specific impacts of maternal obesity, including sex‐specific differences in terms of the epigenetic impact. Male mice of HFD‐fed mothers had 36 dysregulated miRNAs compared to only 1 dysregulated miRNA in females [181]. Similarly, sex‐specific alterations in DNA methylation patterns have also been found in the frontal cortex and hippocampus of young adult rats of HFD‐fed mothers, alongside changes to ASD‐related gene expression, with male offspring affected to a greater extent than females [134]. A more pronounced effect on brain gene expression was also noted in male embryos of HFD‐fed dams, with 386 genes dysregulated in males compared to only 66 genes in females. Interestingly, the genes affected were also largely sex‐specific and nonoverlapping [181], supporting differential mechanistic impacts in males and females.

The placentas of male fetuses also appear to be more vulnerable to the damaging effects of maternal obesity [182], with placental inflammation reported to be more pronounced in the placentas of male fetuses [177]. Similarly, placental BDNF signaling was also decreased more in male fetuses than in female fetuses in mothers with obesity [180].

To date, most of these mechanistic studies suggest that male offspring are more sensitive to the adverse effects of maternal obesity on the brain than female offspring, which aligns with the generally observed increased incidence of neurodevelopmental and cognitive disorders in male offspring. However, the data suggest that female offspring may be more sensitive to the affective impact of maternal obesity [71], with the sex‐specific mechanisms involved yet to be elucidated. Further research is still needed on the underlying mechanisms behind the sex‐specific effects of maternal obesity.

### 4.2 Paternal Mechanisms of Impact

#### 4.2.1 Epigenetic Mechanisms

The main mechanism proposed for the transgenerational inheritance of paternal obesity is via epigenetic changes in the sperm. The impact of paternal diet and obesity on sperm epigenetics is supported by the observation that the methylation status of 1509 genes was changed in human sperm 1 week after bariatric surgery, increasing to 3910 at 1 year postsurgery, highlighting the dynamic regulation of sperm gene methylation and its sensitivity to metabolic change [183]. Similar to the changes seen in maternal obesity, there is also an association between paternal BMI and the hypomethylation of offspring DNA collected from newborn placental cord blood. This hypomethylation persisted in the blood of children up to 7 years old [184], supporting a stable, long‐term impact. Altered methylation patterns have also been detected in other studies, with both hypo‐ and hypermethylation observed depending on the genes studied [185, 186]. For example, hypomethylation of the insulin‐like growth factor 2 (IGF2) promotor in newborns is associated with paternal obesity, and a functional impact of this epigenetic change was confirmed in terms of increased circulating IGF2 levels in children born to fathers with obesity [186, 187]. IGF2 has a key role in growth and development, including in brain development, and IGF2 hypomethylation is associated with higher BMI in children [188, 189].

Diet‐induced histone modifications of sperm [190], have also been reported. In fact, altered H3K4me3 levels, an “activating” histone modification, have been shown in obesity, leading to changes in the regulation of sperm genes involved in metabolism, development, and inflammation. Notably, these alterations are also present in the placenta and embryo, supporting the conservation of these modifications in the offspring, at least during early development [191, 192].

The sncRNA content of sperm is also altered in males living with obesity. The miRNA content of sperm is altered in response to a HFD in both human and animal studies [183, 193, 194], and these may have transgenerational effects. For example, miRNA sperm alterations, specifically in miRNA let‐7c, were shown to be passed down into the epigenome of tissue taken from mouse offspring of HFD fathers [195]. The ability of these miRNAs to directly program phenotypes in the offspring is also supported. Injection of miR19b, an miRNA found to be increased in the sperm of HFD‐fed fathers, into one‐cell embryos induced metabolic changes similar to those seen in offspring from HFD fathers [196]. Another form of ncRNA implicated in this transmission is tsRNAs. tsRNA levels are increased in mouse sperm within 2 weeks of the addition of sugar to the diet [197], supporting rapid dietary modulation of sperm tsRNAs. These sperm tsRNAs can modify gene expression and the metabolic status of the offspring, as direct injection of tsRNA fragments derived from the sperm of HFD males into zygotes produced offspring with markers of metabolic disorder and altered gene expression within embryos, similar to the changes seen in the offspring of HFD males [198]. Moreover, deletion of the tRNA methyltransferase DNMT2 alters levels of tsRNAs in the sperm and prevents paternal HF‐induced metabolic dysfunction in mouse offspring [199]. Recent findings have also indicated a putative role of sperm‐derived mitochondrial‐encoded tRNAs (mt‐tRNAs) and their fragments (mt‐tsRNAs) as a novel epigenetic mechanism by which fathers with obesity may influence offspring metabolic health [200]. Interestingly, a recent study in which hypothalamic Agouti‐related peptide (AgRP) neurons were chemogenetically stimulated in male mice resulted in changes in the fragment size of tsRNA in the sperm that were similar to changes induced by high‐fat feeding, suggesting a putative upstream role of AgRP neurons in paternal HF‐induced metabolic dysfunction [201].

Overall, these data suggest that diverse epigenetic mechanisms in sperm are impacted by paternal diet, HFD consumption, and obesity itself. These are likely key mechanisms by which paternal health directly impacts the offspring. To date, the focus has largely been on metabolic outcomes, some of which are likely to have a neurological impact. However, further research is needed to elucidate the contribution of these mechanisms to neurodevelopmental and cognitive outcomes in the offspring.

#### 4.2.2 Inflammation

Paternal obesity has also been tentatively linked to increased brain inflammation in offspring, although far less research has been undertaken on this topic. A mouse model of paternal obesity found increased inflammatory cytokine (TNF‐α and IL‐6) levels in the hypothalamus of offspring from obese fathers [202]. However, a human study of systemic inflammation biomarkers found no association with paternal obesity [203], while another study found no impact of either maternal or paternal obesity when considered independently. An altered inflammatory biomarker profile was found in children where both parents were obese [204]. Interestingly, similar effects were also seen in a study in mice characterizing hypothalamic inflammation, where combined parental obesity was found to exacerbate the observed pro‐inflammatory effects [202]. This may suggest a more additive effect of paternal obesity toward inflammation in the offspring brain, with more subtle effects potentially observed in comparison to maternal obesity, and additive effects of joint parental obesity, although more research is needed into this interaction before more concrete claims can be made.

#### 4.2.3 Neuronal and Brain Changes

As discussed previously, impaired hippocampal neurogenesis has also been seen in a mouse model of paternal obesity, in association with impaired hippocampal‐dependent learning and memory [119]. As seen in maternal obesity, reduced levels of BDNF have also been observed in the brains of offspring born to HFD‐fed fathers, as well as reduced levels of its receptor TrkB and downstream signaling molecules [119]. Despite these findings, however, studies characterizing the broader molecular and cellular impact of paternal obesity on the brain are currently lacking.

#### 4.2.4 Placental Alterations

Interestingly, and somewhat surprisingly, placental deficits do not appear to be restricted to cases of maternal obesity. Placental defects have also been seen in rodent models of paternal obesity, including impaired placental vascularization and hypoxia [205]. Another study also found changes in placental gene methylation and expression in the embryos of a rodent model of paternal obesity, with effects that were also specific to the sex of the fetus [206]. Female offspring may be more sensitive to these effects, as more pronounced changes to placental vascularization [205] and increased global DNA methylation [206], compared to controls and male offspring, have been reported.

#### 4.2.5 Sex Differences

A number of studies have reported effects from paternal obesity that impact female offspring more severely than males. One of the most marked sex‐specific effects of paternal HFD reported in rats is beta‐cell dysfunction, with this dysfunction and the transcriptomic changes in pancreatic islets being more pronounced in female offspring than in males [207, 208]. In addition, placental changes, including methylation states and vascularization, are also reported to be more severe in female fetuses [205, 206]. A similar effect is seen in terms of gene methylation patterns in fetal cord blood, again with female offspring being more affected [186]. While this suggests that female offspring may be more sensitive to the effects of paternal obesity, this is yet to be characterized in the context of brain function and development. More research is needed into the potential sexually dimorphic mechanisms of paternal HFD, and the resultant neurological phenotypes, particularly as many studies in the field have only considered one offspring sex.

## 5 Conclusion

In conclusion, there is strong evidence of a transgenerational impact of both maternal and paternal obesity on offspring health, including altered brain development, increased risk of neurodevelopmental disorders, and cognitive and behavioral changes. The exact mechanisms behind these associations are not yet known, although emerging evidence supports a role for epigenetic changes including alterations in DNA methylation and ncRNA content, alongside placental alterations and changes in neurotrophic factor signaling in the offspring. The biological sex of the offspring also appears to be important, with male offspring seeming to be more affected by maternal obesity and female offspring potentially more affected by paternal obesity. However, more work is required to further clarify this potential effect in the context of brain health. While a considerable body of work has been undertaken in maternal obesity, several important open questions remain, including the key mechanisms involved. For example, additional studies are needed to further understand the impact of specific dietary components on offspring brain function, and to disentangle how the timing of high‐fat feeding, be that before pregnancy, throughout pregnancy and lactation, or solely during gestation and/or lactation affects brain development and maturation. In addition, future research needs to be dedicated to elucidating the common and divergent physiological and molecular events that occur in relation to the different obesity measurements utilized in human maternal studies, such as prepregnancy high BMI and gestational weight gain, and the resultant impact on offspring neurodevelopmental outcomes.

Moreover, there is a paucity of data on the impact of paternal obesity on neurological outcomes and the mechanisms involved. In general, more paternal diet manipulation studies are needed, as well as potential investigation of changes to sperm epigenetics in response to a HFD. While there are certainly further studies required, the emerging data support a significant impact of paternal obesity on the offspring brain and associated behaviors. These studies are essential given the increasing global burden of obesity. Understanding the relationship between parental obesity and offspring neurodevelopmental outcomes, and the mechanisms involved, could inform the advice given to people trying to get pregnant to improve their preconceptual health. This could include dietary manipulation, such as supplementation with polyunsaturated fatty acids, vitamin D and folate [158, 209], weight loss, or novel miRNA targeted therapies [145]. In addition, identifying children of mothers or fathers with obesity who may be at greater risk of developing neurodevelopmental and related disorders may provide an opportunity to implement interventional educational, linguistic, social, and exercise strategies to improve offspring brain health across the life course [210, 211, 212].

## Funding

Lancaster University, 10.13039/100010029.

## Conflicts of Interest

The authors declare no conflicts of interest.

## Supporting information


**Table S1:** A summarization of studies exploring the connection between maternal or paternal BMI and neurodevelopmental outcomes seen in offspring.
**Table S2:** A summarization of studies exploring the connection between maternal or paternal BMI and the behavioral and emotional outcomes seen in offspring.
**Table S3:** A summarization of studies exploring the connection between maternal or paternal BMI and learning and memory outcomes seen in offspring.

## Data Availability

Data sharing not applicable to this article as no datasets were generated or analysed during the current study.
