# Supplementary material for: A Review of the Effects of Maternal and Paternal Obesity on Neurodevelopmental Disorders and Related Neurobiology in Rodent and Human Offspring
Source: Obes Rev. 2025 Dec 17;27(5):e70067. doi: 10.1111/obr.70067 (PMC13070895; doi:10.1111/obr.70067)
Supplement: Supplementary file 1 — Table S1: A summarization of studies exploring the connection between maternal or paternal BMI and neurodevelopmental outcomes seen in offspring. Table S2: A summarization of studies exploring the connection between maternal or paternal BMI and the behavioral and emotional outcomes seen in offspring. Table S3: A summarization of studies exploring the connection between maternal or paternal BMI and learning and memory outcomes seen in offspring. [file OBR-27-e70067-s001.docx]

**Supplemental Tables**

**Supplementary Table 1: A summarisation of studies exploring the connection between maternal or paternal BMI and neurodevelopmental outcomes seen in offspring.**

| **Maternal** | | | | | | |
| --- | --- | --- | --- | --- | --- | --- |
| Topic | Subject | Offspring  age | Cohort size | Results | Interpretation | Reference |
| ADHD | Human | 7-8 and 10-12 years old | 12,556 children | - Teacher rated hyperactivity and inattention in children - Children of mothers that were overweight or obese and gained a large amount of weight during pregnancy had a twofold increase in risk of ADHD symptoms | Maternal pre-pregnancy BMI is associated with ADHD symptoms in children | ^43^ |
| ADHD | Human | 5 years | 1,714 | - Maternal obesity or overweight was associated with high ADHD scores and emotional difficulty in mothers’ and teachers’ assessment of children’s ADHD symptoms and emotionality | Maternal pre-pregnancy BMI is associated with ADHD and emotional regulation | ^44^ |
| ADHD | Human | Mean age 7.3 | 174 children | - Maternal pre-pregnancy BMI was significantly associated with ADHD symptoms and impaired executive function | Maternal pre-pregnancy BMI has an impact on child ADHD risk, and may mediate this effect through impaired executive function | ^46^ |
| ADHD | Human | Born 1992 -2000, followed until diagnosis or until end of 2009 | 673,632 | - An association between maternal BMI and offspring ADHD was observed - Sibling comparison attenuated this association | The association observed between maternal BMI and offspring ADHD may be explained by familial confounding | ^47^ |
| ADHD | Mice | 4-5 months | N=9 obese offspring  N=8 control | - Male offspring of obese dams were more active than control mice in tests of hyperactivity | Maternal obesity has a direct link to hyperactivity in the offspring | ^48^ |
| ASD | Human | 2-5 years | 1,004 | - Children with ASD or developmental disorders were more likely to have mothers that had experienced metabolic conditions during pregnancy versus control - Metabolic conditions - Diabetes, obesity or hypertension during pregnancy | Maternal metabolic conditions may be associated with neurodevelopment problems in children | ^29^ |
| ASD | Human | 2 years | 62 mother-offspring dyads | - Obese versus non-obese mothers of preterm children - Maternal obesity was associated with a higher risk of ASD and developmental delay at 2 years old | Preterm infants may have a higher chance of poor neurodevelopment when born to obese mothers | ^30^ |
| ASD | Human | Followed from birth to 5 years | 4,800 | - Obese or very underweight mothers had increased incidence of very low birth weight - Children of these mothers also had over twice the likelihood of ASD | Underweight or obese mothers indirectly increased risk of ASD in children via low birth weight | ^38^ |
| ASD | Human | Median age 6 years | 2,734 | - Combination of maternal pre-pregnancy obesity and diabetes correlated with ASD and developmental disorders in children - Slight increase in the incidence of ASD in offspring of obese mothers without diabetes | Maternal obesity may increase the risk of children with ASD | ^31^ |
| ASD | Human | Births from 2006 onwards | 40,846 | - Maternal obesity increased the risk of a child having ASD by 1.5-fold. - For mothers with both gestational diabetes and obesity, the association was twofold for having a child with ASD compared with controls. | Maternal obesity and gestational diabetes may cause higher risk of ASD | ^32^ |
| ASD | Human | Children born between 1993 - 2008 | 4,419 children | - The association between maternal pre-pregnancy BMI and ASD risk was non-linear and J-shaped - The adjusted odds ratio for maternal obesity and child ASD was 1.54 | Extremes of maternal BMI was linked to a modestly higher risk of ASD – being obese or underweight | ^37^ |
| ASD | Human | 8 years | 26,108 | - ASD risk was significantly associated with higher maternal pregnancy weight gain, but not maternal pre-pregnancy BMI | Gestational weight gain may be linked to ASD risk | ^26^ |
| ASD | Human | 1-17 years | 129,733 births | - Maternal pre-pregnancy weight of 90kg or more was associated with a higher risk of ASD in children compared to control - Gestational weight gain of 18kg or more was also associated with higher ASD risk | Maternal pre-pregnancy obesity and high gestational weight gain may be independent risk factors for ASD | ^34^ |
| ASD | Human | 4-27 years old | 333,057 | - Higher maternal pregnancy baseline BMI was associated with higher ASD risk - However, after sibling analysis it was concluded this association may be due to familial confounding - Excessive GWG was suggestive of elevated risk of ASD | The association seen between maternal BMI and ASD may be a proxy for more genetic factors, but some evidence for effect of excess GWG | ^28^ |
| ASD | Human | 2-5 years old | 4,409 mother-child dyads | - Maternal pre-pregnancy BMI categorised as underweight, normal, overweight, obesity class 1 and obesity class 2/3 - Maternal obesity class 2/3 was associated with higher ASD and developmental disorder incidence | Maternal severe obesity is associated with increased risk of ASD and developmental disorder | ^33^ |
| ASD | Human | 2-9 years | 2,941 children | - Maternal pre-pregnancy BMI and gestational weight gain - Excessive gestational weight gain was associated with increased risk of child ASD in overweight and obese mothers - The associated between maternal pre-pregnancy BMI and ASD risk was not significant | Excessive gestational weight gain may increase risk of ASD in already overweight or obese mothers | ^27^ |
| ASD and ADHD | Human | Average age 13.3 years | 81,892 children | - Risk of having a child with ADHD or ASD increased if the mother was overweight or obese. The risk of ASD also increased if the mother was underweight | Maternal BMI increases the risk of ASD and ADHD | ^36^ |
| Intellectual disability | Human | 3-6 years | 78,675 mother-child pairs | - Risk of intellectual disability was increased for children of obese mothers, and was highest for children of morbidly obese mothers (OR=1.74 for severe intellectual disability) | Pre-pregnancy obesity may be a risk factor for intellectual disability in children | ^91^ |
| Intellectual disability | Human | Children born between 1992 - 2006 | 467,485 children | - Mothers’ gestational weight gain and whether children had been diagnosed with Intellectual developmental disorders (IDD). - Inadequate GWG was associated with increased risk of IDD independent of maternal pre-pregnancy BMI - Extremely excessive GWG were associated with increased risk of IDD, but only in mothers already with a high pre-pregnancy BMI | High and low maternal gestational weight gain may be linked with increased likelihood of intellectual developmental disorders. | ^80^ |
| Schizophrenia | Human | 13-21 years at start of study and 30-38 at end of follow-up | 6,633 | - Maternal pre-pregnancy BMI and offspring schizophrenia diagnosis - Maternal BMI over 30 was significantly associated with an increased incidence of schizophrenia and spectrum disorders in offspring | High pre-pregnancy BMI may increase the risk of offspring schizophrenia | ^53^ |
| Schizophrenia | Human | 0-45 years old at start, 17-67 when follow up ended | 68,571 mother child dyads | - Maternal BMI measured at mean 15.7 gestational weeks - Two time periods, children born 1950-74, or born 1975-99 - In 1950-74 cohort, maternal underweight predicted mental disorder - In 1975-99 cohort, severe obesity predicted mental disorder and schizophrenia spectrum disorder | Maternal BMI can impact the likelihood of child mental disorder, which was time dependent | ^56^ |
| Schizophrenia | Human | Mean 18.9 years for patients and 20.2 years for controls | 336 people | - Maternal early and late pregnancy BMI - Early pregnancy – one unit increase in BMI correlated with 24% increased risk of schizophrenia - Late pregnancy – one unit increase in BMI correlated with 19% increased risk of schizophrenia | High maternal BMI correlated with increased risk of schizophrenia, especially during early pregnancy | ^57^ |
| Social behaviour | Mice | 7-8 weeks | N=14 per group | - Social behaviour tested at 7-8 weeks old - Offspring of mothers fed a high fat diet showed impaired sociability, with fewer reciprocal interactions and no social novelty preference | Maternal obesity effects the social behaviour of offspring | ^40^ |
| **Paternal** | | | | | | |
| ASD | Human | 4.0-13.1 years, mean 7.4 | 92,909 | - Paternal obesity was significantly associated with an increased risk of ASD and Asperger’s disorder | Paternal obesity may be a risk factor for ASD | ^23^ |
| ASD and ADHD | Human | 12 years | 2,666 | - Paternal obesity was associated with a two-fold risk of ADHD. The prevalence of ASD was too low for analysis | Increased paternal BMI was a risk factor for ADHD | ^24^ |
| **Both parents** | | | | | | |
| ADHD | Human | 7-8 years | 1,915 | - High maternal pre-pregnancy BMI was associated with ADHD, hyperactivity and inattention problems. Paternal BMI was not associated with outcomes | Maternal BMI has an impact on risk of ADHD and behavioural problems  Paternal BMI had no impact | ^45^ |
| ADHD | Human | 0-7 years old | 38,314 children | - Maternal and paternal pre-pregnancy BMI - Strength and difficulties questionnaire completed by parents when child was 7 years old - Maternal and paternal BMI were associated with behavioural difficulties, including hyperactivity, and emotional and conduct issues | Parental obesity is associated with offspring behavioural issues, including hyperactivity | ^25^ |
| ASD and ADHD | Human | 5 years | 1,827 | - Child cognitive and psychomotor development, ASD and ADHD prevalence tested against maternal and paternal BMI - There was no significant difference to controls for ASD symptoms. There was association with ADHD symptoms for both maternal and paternal BMI | Maternal obesity does not increase the risk of ASD, but it does increase risk of ADHD | ^39^ |
| Development | Human | 4-36 months old | 3759 singletons and 1062 nonrelated twins | - Ages and stages questionnaire filled in by parents when children were 4,8,12,18,24,30 and 36 months old - Results were compared with parental factors - Children of obese mothers were more likely to fail the fine motor skills domain - Paternal obesity correlated with failure of the personal-social domain | Parental obesity was associated with childhood development delays, specific to each parent | ^111^ |

**Supplementary Table 2: A summarisation of studies exploring the connection between maternal or paternal BMI and the behavioural and emotional outcomes seen in offspring.**

| Topic | Subject | Offspring  age | Cohort size | Results | Interpretation | Name of study |
| --- | --- | --- | --- | --- | --- | --- |
| **Maternal** | | | | | | |
| Anxiety | Mice | 3-month and 12-month-old | N = 8 to 12 per condition | - Offspring tested in elevated plus maze, forced swim test, chronic social defeat stress, open field and social avoidance - Less open arm entries in the elevated plus maze in 12-month-old HFD offspring that was not evident at 3-months old. | Maternal obesity causes long lasting delayed anxiety-like behaviours in the offspring | ^65^ |
| Anxiety | Mice | 90 days old | N = 21-22 per group | - HFD offspring displayed more anxiety-like behaviour in the elevated plus maze and the food neophobia test compared to control offspring | Maternal HFD correlated with increased anxiety related behaviour | ^64^ |
| Anxiety | Non-human primates | Post natal day 130 | N = 4 to 12 per group | - Novel object exposure at post-natal day 130 - 55% of HFD female offspring displayed increased anxiety in novel object test - HFD male offspring displayed increased aggression - 78% of HFD displayed aberrant behaviour (anxiety/aggression) during behavioural testing compared to 11% of control offspring | Maternal diet influenced offspring anxiety-like behaviour | ^71^ |
| Anxiety | Non-human primates | 11 months | N = 12-24 per group | - Offspring exposed to a maternal HFD showed increased anxiety-like behaviour, increasing the number of vocalisations and the occurrences of anxious behaviours. | Maternal HFD exposure leads to behavioural abnormalities in offspring, including increased anxiety | ^72^ |
| Anxiety | Rats | 90 days | N = 11-14 per group | - HFD offspring showed increased anxiety related behaviour in elevated plus maze, light-dark transition and the open field test compared to controls | Maternal HFD is related to offspring increased anxiety behaviour | ^67^ |
| Anxiety | Rats | 56 days | N = 10 per group | - HFD offspring displayed more anxiety like behaviour in the elevated plus maze than control offspring. | Maternal HFD led to anxiogenic offspring phenotype | ^66^ |
| Anxiety and depression | Mice | 12 weeks | N = 8 per group | - Results of the open field test and elevated plus maze indicated HFD offspring showed more anxious behaviour than controls - HFD offspring also showed more depressive like behaviour in the forced swim test | Maternal HFD is associated with anxiety and depressive-like behaviour in the offspring | ^73^ |
| Depression | Rats | 28-69 days | N = 10 per group | - Offspring of maternal HFD had increased immobility time and decreased swimming in the forced swim test | Maternal HFD is associated with a depressive-like phenotype in offspring | ^42^ |
| Anxiety, Sociability, ADHD | Mice | 5.5-6 weeks | N= 7-30 per group | - Maternal HFD resulted in increased anxiety and decreased sociability in female offspring at 5.5-6 weeks of age - HFD male offspring showed increased hyperactivity | Maternal HFD impacts the behaviour of the offspring | ^49^ |
| Behaviour | Human | 5 years | 4,094 mother – child pairs | - Mothers and teachers completed the strengths and difficulties questionnaire - Higher maternal pre-pregnancy BMI correlated with increased child behavioural problems, peer relationship problems and a decrease in cognitive flexibility | Maternal pre-pregnancy obesity is linked to increased behavioural problems in children | ^77^ |
| Behaviour | Human | 5-12 years | 469 mother – child pairs | - Mothers completed the child behaviour checklist and teachers completed a teacher report form on the behaviour of the child - Pre-pregnancy maternal obesity was associated with an increase in child behaviour problems in both mother and teacher reports | Maternal pre-pregnancy obesity is linked to increased behavioural problems in children | ^75^ |
| Behaviour | Human | 5-17 years old | 2785 | - Maternal pre-pregnancy BMI - Children internalising and externalising problems at ages 5, 8, 10, 14, and 17 - There was a significant association between maternal BMI and internalising problems and a positive association with externalising problems | Maternal pre-pregnancy obesity may influence behavioural and emotional issues in offspring | ^74^ |
| Behaviour | Human | Pregnancy to 10 years old | 511 mother-infant dyads | - Gestational weight gain and pre-pregnancy BMI - Internalising and externalising behaviours and attention problems exhibited a small increase in children of mothers with obesity compared to normal weight mothers. | Maternal pre-pregnancy obesity was associated with a small increase in behavioural problems | ^76^ |
| Mental health disorders | Human | Birth to 18 years | 38,211 mother offspring pairs | - Maternal weight status and the number of offspring visits to a physician for mental health disorders, - Maternal obesity correlated with significantly more physician visits for mental health conditions, mood and anxiety disorders, ADHD than control children | Maternal obesity correlates with increased presentation of mental health disorders in offspring via health care. | ^62^ |
| Social behaviour, anxiety | Mice | 13 weeks | Total 127 offspring | - High fat and sugar diet offspring tested on elevated Plus maze, novel object recognition, open field and social preference - No difference in offspring locomotion or social behaviour. - High fat and sugar offspring displayed reduced anxiety behaviour - Males were impaired in novel object recognition | Maternal diet effects the behaviour of offspring with potential sex specific differences | ^105^ |
| Depression | Human | 5-17 years | 2,868 children | - Children followed up at ages 5, 8, 10, 14 and 17. - Child behaviour checklist given to primary caregiver to assess for affective disorders in children - There was a higher risk of affective disorders in children of overweight or obese mothers between ages 5-17 (fully adjusted OR for obese mothers = 1.72) | Maternal pre-pregnancy overweight and obesity may be a risk factor for the development of affective disorders in their children in later life | ^63^ |
| **Paternal** | | | | | | |
| Anxiety | Rat | 32 days | N = 6-13 per sex per group | - Increase in attack and defensive behaviour in HFD offspring - HFD offspring displayed more anxious behaviour | Paternal obesity alters behavioural responses and increases anxiety-like behaviour of offspring | ^116^ |
| **Both parents** | | | | | | |
| Behaviour | Human | 0-8 years | ~7,500 | - No association of maternal pre-pregnancy weight with externalising/internalising behaviour problems, non-verbal skills or attention deficits was found. - Paternal overweight was also not associated with any behavioural issues | There is little evidence of intrauterine effect of pre-pregnancy weight on cognitive function and behaviour of children | ^78^ |
| Behaviour | Human | 0-7 years old | 38,314 children | - Maternal and paternal pre-pregnancy BMI - Strength and difficulties questionnaire completed by parents when child was 7 years old - Maternal and paternal BMI were associated with behavioural difficulties, including hyperactivity, and emotional and conduct issues | Parental obesity is associated with offspring behavioural issues | ^25^ |

**Supplementary Table 3: A summarisation of studies exploring the connection between maternal or paternal BMI and learning and memory outcomes seen in offspring.**

| Topic | Subject | Offspring age | Cohort size | Results | Interpretation | Name of study |
| --- | --- | --- | --- | --- | --- | --- |
| **Maternal** | | | | | | |
| Cognitive performance | Human | 5 and 7 years | N = 11,025 at 5 years old  N = 9,882 at 7 years old | - UK mother and child cohort with data on children at ages 5 and 7. Children were subject to standard cognitive assessment - Maternal overweight/obesity was negatively correlated with child cognitive performance - The effect size was modest (10-point increase in maternal BMI, decrease in cognitive performance of around 1/10^th^ standard deviation at age 7) - The relationship strengthened with age | Maternal BMI is negatively correlated with child cognitive performance | ^83^ |
| Cognitive performance | Human | 60-83 months | 3,412 | - Children underwent Peabody individual achievement reading recognition and mathematic tests to evaluate cognition - Offspring of obese mothers had lower scores, an average of 3 points for reading and 2 for mathematics | Maternal obesity negatively impacts child cognitive test scores | ^82^ |
| Cognitive performance | Human | 9 to 11 years old | 2,084 | - Children’s verbal recognition and perceptual reasoning were assessed at 9 years old - Children of mothers with obesity had lower verbal recognition scores, though perceptual reasoning was not affected by maternal weight | Maternal obesity may have long term effects on child cognition | ^84^ |
| Development | Human | 2 years old | 6,850 | - Maternal pre-pregnancy BMI and Child mental and motor development at age 2 - The risk of delayed mental development was higher among children of obese or underweight mothers | Maternal low or high pre-pregnancy BMI was associated with risk of delayed mental development | ^79^ |
| Development | Human | 3 years old | 382 dyads | - Maternal pre-pregnancy BMI and child performance in the Psychomotor Development Index (PDI) and Mental Development Index (MDI) - Maternal obesity was associated with lower PDI scores in male children, but not female - No association was seen with MDI | A sex specific association was seen between maternal obesity and psychomotor development | ^85^ |
| IQ | Human | 7 years old | 30,212 children | - Child IQ measured at 7 years old by Wechsler Intelligence Scales - Maternal pre-pregnancy obesity was associated with reduced IQ compared to children of normal weight women - Obese women who gained excessive weight during pregnancy increased this association | Maternal obesity was associated with reduced child IQ, and this was further accelerated by excessive GWG | ^86^ |
| IQ | Human | Early pregnancy to 10 years old | 530 mother-infant dyads | - Pre-pregnancy height and weight recorded, as well as gestational weight gain - Child IQ and executive function measured at age 10 - Maternal obesity was associated with reduced IQ and executive function. - Increased GWG was associated with reduced executive function but was not associated with IQ. | Maternal obesity has a stronger relationship with child IQ and executive function than GWG | ^88^ |
| IQ and cognition | Human | 6.5 and 16 years old | 11276 children | - Maternal BMI at 35-weeks gestation - Child IQ measured at 6.5y, and neurocognition assessed at 16y - Higher maternal BMI was associated with generally lower IQ results, as well as lower neurocognitive scores | Maternal obesity may be associated with poorer offspring brain development | ^89^ |
| IQ | Human | Around 5 years old | 1100 children | - Child IQ measured at around 5 years old by Wechsler Intelligence Scales - Pre-pregnancy obesity was associated with a decrease in verbal and performance IQ domains compared to normal BMI mothers | Higher pre-pregnancy BMI is associated with altered child cognitive development | ^90^ |
| Learning and memory | Mice | 20 weeks | N = 9-10 per group | - Male offspring of HFD mothers tested in Morris Water Maze - Offspring fed control diet were similar to control mice. Offspring fed high fat diet had worse retention in Morris water maze | Maternal high fat diet ‘sensitises’ offspring to effects of high fat diet | ^92^ |
| Learning and memory | Rat | 110-118 days | N = 12 per group | - Morris water maze and open field testing - High fat offspring took longer and swam farther to find the platform during learning phase of MWM. This was not impacted by postweaning diet | Maternal HFD impacted offspring spatial learning and memory negatively | ^93^ |
| Learning and memory | Mice | 13 weeks | Total 127 offspring | - High fat and sugar diet offspring tested on elevated Plus maze, novel object recognition, open field and social preference - No difference in offspring locomotion or social behaviour. - High fat and sugar offspring displayed reduced anxiety behaviour - Males were impaired in novel object recognition | Maternal diet effects the behaviour of offspring with potential sex specific differences | ^105^ |
| Learning and memory | Mice | 12 weeks | N = 8-9 per group | - Y-maze and novel object recognition at 12 weeks - There was a significant decrease in the time spent exploring the novel object in male mice offspring of HFD dams compared to control, but no difference in female offspring | Maternal high fat diet may cause difference in attention and cognitive function in offspring and males may be more susceptible to this effect | ^104^ |
| Learning and memory | Mice | 4 and 12 weeks | N = 8-10 per group | - Barnes maze was performed by different populations of 4-week and 11-week-old mice - Latency to escape and distance covered was significantly longer in HFD offspring than control in 4-week-old mice - There was no difference to control in performance at 11-weeks | Maternal HFD negatively affected spatial memory in young offspring, but not adult | ^100^ |
| Learning and memory | Rats | 10 weeks | N = 9-11 per group | - Male pups of HF, highly palatable (HP) and control diet assessed for visual discrimination and serial reversal learning - Reversal learning was impaired in HFD offspring | Maternal high fat diet can result in cognitive inflexibility | ^99^ |
| Learning and memory | Rat | 95 days | N = 7-9 per group | - Male offspring of dams fed a HFD were assessed using the novel object test and the Barnes maze - Male offspring were found to have impaired recognition of novel objects, as well as slower learning and longer escape latency in the Barnes maze | Offspring of HFD fed mothers had impaired learning and spatial memory | ^96^ |
| Learning and memory | Mice | 35 days, 90 days, 18 months | N = 11-16 per group | - HFD and control offspring of three different ages underwent behavioural testing - Reference memory was seen to be impaired in HFD offspring in the T-maze task by 90 days - By 18 months working memory tested by the Y-maze was also impaired in HFD offspring - No difference was seen at 35 days between control and HFD offspring behaviour | Maternal HFD offspring are at increased risk of cognitive deficit | ^102^ |
| **Paternal** | | | | | | |
| Learning and memory | Mice | 8 weeks | N = 16-21 per group | - Cognitive impairment was seen in the paternal HFD offspring during behavioural tests – impaired hippocampal dependent learning and memory | Paternal obesity can negatively impact the cognitive function of offspring | ^119^ |
| **Both parents** | | | | | | |
| IQ | Human | 5 years old | 1,783 | - Both mother and child underwent IQ tests - Maternal BMI was negatively associated with child IQ, for every unit increase in BMI there was a reduction in BMI of -0.40 points unadjusted, and -0.27 after adjustment - Paternal BMI had a comparable result, with a reduction of -0.26 IQ points per BMI unit | Maternal BMI is associated with child IQ, but as paternal is similarly associated this may not be pregnancy specific | ^117^ |
| IQ | Human | 8 years old | 14,541 | - Child IQ measured at 8 years old via the Wechsler Intelligence scale for children-III - Maternal and paternal pre-pregnancy obesity was weakly associated with child IQ performance, with maternal having a stronger effect | Maternal obesity may have a weak biological effect on child IQ | ^118^ |
| Learning and memory | Rats | 4 months | N = 9-12 per group | - The performance of offspring of HFD mothers and fathers in the novel object recognition test was compared to control - Rats born to obese parents showed impaired memory during testing - Maternal obesity led to short (2h) and long term (24) deficits, whereas paternal obesity only showed short term deficits. | Maternal and paternal obesity impact the encoding stage of memory, but maternal obesity also impairs long term memory | ^95^ |
